# Datanator: an integrated database of molecular data for quantitatively modeling cellular behavior

**DOI:** 10.1093/nar/gkaa1008

**Published:** 2020-11-11

**Authors:** Yosef D Roth, Zhouyang Lian, Saahith Pochiraju, Bilal Shaikh, Jonathan R Karr

**Affiliations:** Icahn Institute for Data Science and Genomic Technology and Department of Genetics and Genomic Sciences, Icahn School of Medicine at Mount Sinai, 1255 5th Avenue, Suite C2, New York, NY 10029, USA; Icahn Institute for Data Science and Genomic Technology and Department of Genetics and Genomic Sciences, Icahn School of Medicine at Mount Sinai, 1255 5th Avenue, Suite C2, New York, NY 10029, USA; Icahn Institute for Data Science and Genomic Technology and Department of Genetics and Genomic Sciences, Icahn School of Medicine at Mount Sinai, 1255 5th Avenue, Suite C2, New York, NY 10029, USA; Icahn Institute for Data Science and Genomic Technology and Department of Genetics and Genomic Sciences, Icahn School of Medicine at Mount Sinai, 1255 5th Avenue, Suite C2, New York, NY 10029, USA; Icahn Institute for Data Science and Genomic Technology and Department of Genetics and Genomic Sciences, Icahn School of Medicine at Mount Sinai, 1255 5th Avenue, Suite C2, New York, NY 10029, USA

## Abstract

Integrative research about multiple biochemical subsystems has significant potential to help advance biology, bioengineering and medicine. However, it is difficult to obtain the diverse data needed for integrative research. To facilitate biochemical research, we developed Datanator (https://datanator.info), an integrated database and set of tools for finding *clouds* of multiple types of molecular data about specific molecules and reactions in specific organisms and environments, as well as data about chemically-similar molecules and reactions in phylogenetically-similar organisms in similar environments. Currently, Datanator includes metabolite concentrations, RNA modifications and half-lives, protein abundances and modifications, and reaction rate constants about a broad range of organisms. Going forward, we aim to launch a community initiative to curate additional data. Datanator also provides tools for filtering, visualizing and exporting these data clouds. We believe that Datanator can facilitate a wide range of research from integrative mechanistic models, such as whole-cell models, to comparative data-driven analyses of multiple organisms.

## Graphical Abstract

**Graphical Abstract:**
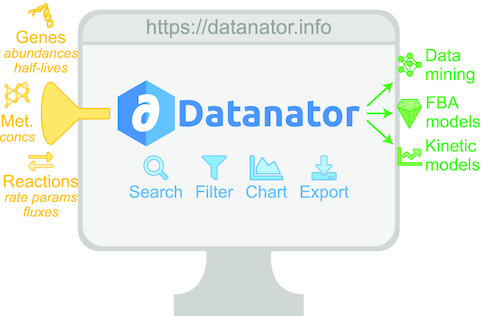
Datanator (https://datanator.info) is an integrated database of molecular data. To help investigators find relevant data for research even in the absence of direct measurements, Datanator includes tools for assembling "clouds" of measurements centered on a specific molecule or reaction in a particular organism that encompass measurements of similar molecules and reactions in similar organisms. Datanator is ideal for integrative and comparative analysis and modeling.

## INTRODUCTION

Integrative research about multiple biochemical subsystems has significant potential to help advance biology, bioengineering and medicine. For example, whole-cell models that account for all of the biochemistry in cells could help scientists conduct experiments in silico, help physicians personalize medicine and help engineers design microbes (1,2).

Currently, it is challenging to obtain the diverse and extensive data needed to analyze multiple subsystems. For example, Resource Balance Analysis (RBA) requires data about the composition of cells and macromolecular complexes, the abundances of enzymes and the maximum rates of reactions (3). Whole-cell models require even more data, such as the concentration of each metabolite, the abundance and turnover of each RNA and protein, and the rate law of each reaction.

New experimental technologies have begun to produce the data needed for integrative research. For example, RNA-seq has enabled transcriptome-wide profiles of RNA abundances and half-lives, tandem mass spectrometry has enabled proteome-scale profiles of protein abundances and half-lives, and liquid chromatography-mass spectrometry has enabled metabolome-scale profiles of metabolite concentrations. Furthermore, this data is increasingly accessible. For example, ArrayExpress contains over 400 000 RNA expression profiles (4).

Several investigators have begun to use this data to conduct more integrative analyses of biochemical networks. For example, Sanchez *et al.* (5) used proteomic data and reaction rate parameters to develop a constraint-based model of the gene expression and metabolism of yeast; Thiele *et al.* (6) used genetic information and other data to build a constraint-based model of the transcription, RNA modification, translation, complexation and metabolism of *Escherichia coli*; Goelzer *et al.* used metabolomic and proteomic data to develop an RBA model of 72 subsystems of *Bacillus subtilis* (3) and we and others used a wide range of data (7) to develop a hybrid model of 28 subsystems of *Mycoplasma genitalium* (8). However, even the most extensive studies have only been able to capture limited facets of the relationship between genotype and phenotype because it is currently challenging to acquire and integrate the data needed for more comprehensive analyses. Encouraged by these successes, we believe that increased access to data would enable investigators to more comprehensively and more deeply investigate the relationship between genotype and phenotype.

Although extensive data is available, obtaining this data is one of the biggest bottlenecks to integrative research. This sentiment was echoed by a recent community survey of the bottlenecks to biomodeling (9). One fundamental barrier is our limited ability to experimentally characterize biochemistry. For example, the most comprehensive metabolomics datasets only capture a small fraction of the metabolome. Long-term, the community must develop new experimental technologies for characterizing molecular biology.

In addition, it is difficult to utilize the existing data: the existing data is siloed in different databases and articles for different types of data; the existing databases use different formats, identifiers, and units (e.g. PAXdb (10) provides data in TSV format, whereas SABIO-RK (11) provides data in SBML format (12)); the APIs to the existing databases have different interfaces and there are limited tools for finding data about similar molecular mechanisms (e.g. concentrations of similar metabolites) when direct measurements are not available. These obstacles make it difficult to compare and integrate data.

We believe that many of these secondary challenges can be addressed with practical computational solutions. To address these challenges, we developed Datanator (https://datanator.info), an integrated database of quantitative molecular data about a wide range of organisms.

To start, we have aggregated several key types of data, including metabolite concentrations, RNA modifications and half-lives, protein abundances and modifications, and reaction rate parameters from several databases and articles. Going forward, we aim to launch a community initiative to curate additional data. To help researchers search, compare and integrate this heterogeneous data, we have normalized the annotation, units, and representation of the data.

On top of this database, Datanator provides tools for finding relevant data for research. Similar to other databases, Datanator provides tools for finding data about specific molecules and reactions in particular organisms and environments. In the absence of direct data, Datanator also provides tools for building *clouds* of data that include chemically-similar molecules and reactions in phylogenetically-similar organisms and similar environments. For example, Datanator can help researchers find data about the concentration of ATP and similar metabolites in *E. coli* or other enterobacteria in M9 or other minimal media. These data clouds can help researchers impute missing information. Datanator also provides tools for filtering these clouds, charting their distribution, and exporting them.

We believe that Datanator can facilitate a wide range of research. For example, Datanator can help investigators find information for comparative analyses of multiple organisms and tissues, multi-dimensional analyses of relationships within biochemical networks, and integrative mechanistic models of multiple subsystems such as whole-cell models.

Here, we describe the content of Datanator and the tools for searching and visualizing this data. In addition, we summarize how we implemented Datanator, compare Datanator to several existing databases, outline the types of research that we believe Datanator can facilitate and discuss how we plan to enhance Datanator.

## INTEGRATED DATABASE OF MOLECULAR DATA

**Figure 1.**
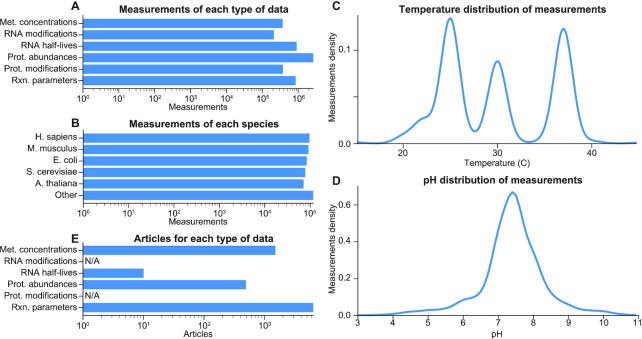
Datanator encompasses several key types of molecular data about a wide range of organisms in a broad range of environments. (**A**) Number of measurements of each type of data. (**B–D**) Distributions of the genotype and environment of the measurements in the database. (**E**) Number of articles that Datanator integrates data from for each type of data.

Because research often requires many types of data, Datanator includes several types of quantitative and categorical data. Currently, this includes 3841 measurements of the concentrations of 1621 metabolites aggregated from ECMDB (13), YMDB (14) and several articles; 589 reconstructed RNA modification profiles aggregated from MODOMICS (15); 75 836 measured RNA half-lives aggregated from several articles; 2 634 941 measurements of the abundance of 846 970 proteins aggregated from PaxDB; 4083 reconstructed protein modification profiles aggregated from PRO (16) and 61 734 measurements of the rate parameters of 37 858 reactions aggregated from SABIO-RK (Figure 1A). In addition, we are working to include nearly 1 000 000 measurements of RNA localizations from RNALocate (17), lncATLAS (18) and one article; over 100 000 measured and predicted protein localizations from eSLDB (19), the Human Protein Atlas (20), PSORTdb (21) and SubCell (22); >50 000 measurements of protein half-lives from nine publications; >200 000 additional measurements of reaction parameters from BRENDA (23); and reaction flux measurements for 36 organisms from CeCaFDB (24).

In total, Datanator currently includes data for 1030 organisms (Figure 1B) across a wide range of environmental conditions (Figure 1C, D) from over 8000 articles (Figure 1E).

## TOOLS FOR SEARCHING THE SEA OF DATA

Datanator also provides unique tools for searching for relevant data for research. Similar to other databases, Datanator provides basic functionality for finding data about specific molecules and reactions in particular organisms and environments. On top of this, Datanator provides tools for assembling *clouds* of related data – ensembles that also encompass measurements of similar molecules and reactions in similar organisms and environments. In the absence of direct measurements, these clouds can help researchers impute missing information. A brief tutorial on how to use these tools is available at https://datanator.info/help.

### Basic searching for specific measurements

**Figure 2.**
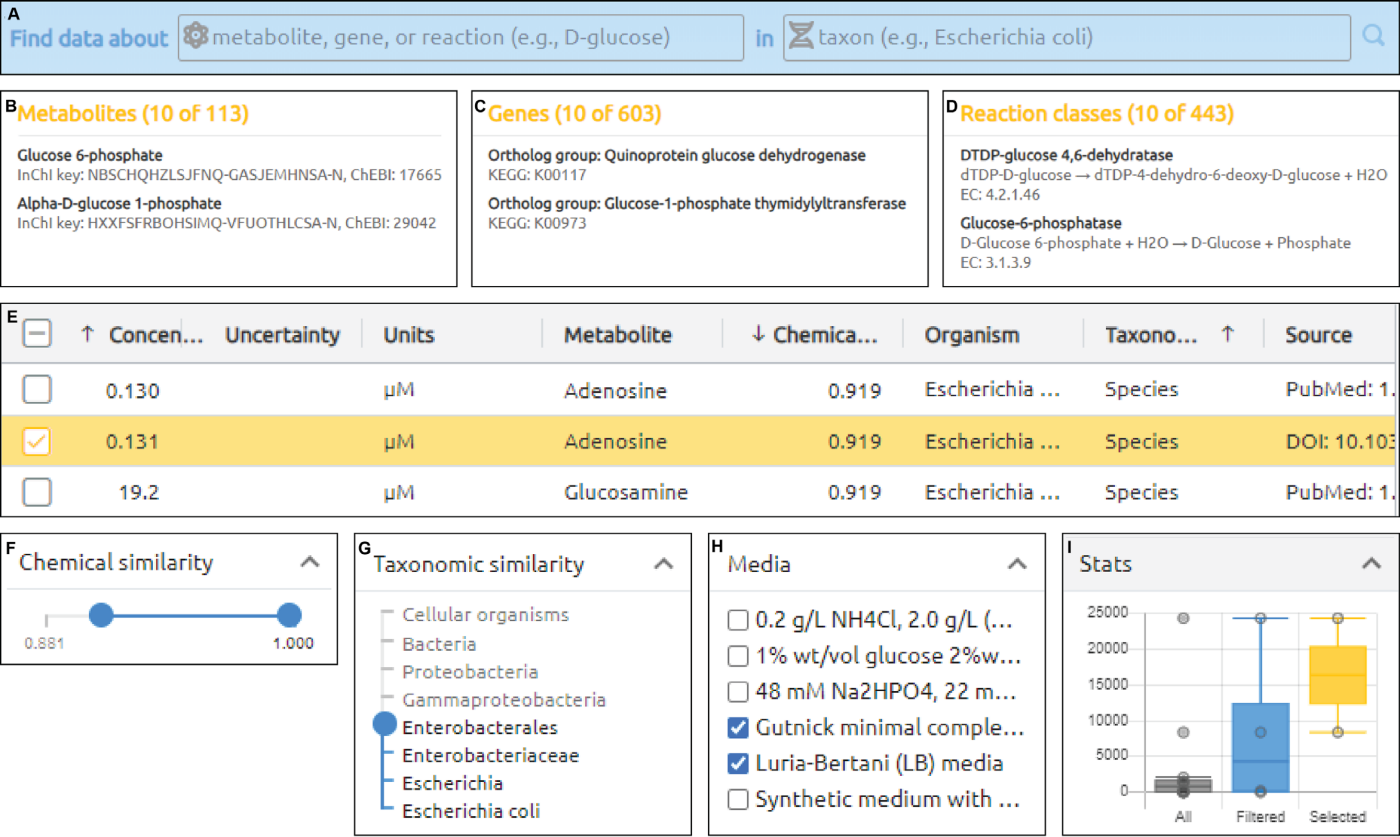
Datanator provides interactive tools for creating and visualizing clouds of data centered on specific molecules and reactions in specific organisms and environments. (**A**) Search form. (**B–D**) Search results grouped by class (metabolites, genes, or reaction). (**E**) Tables of clouds of potentially relevant measurements about a molecule or reaction. (**F–H**) Tools for filtering the data clouds. (**I**) Charts for visualizing the distributions of the data clouds.

Users can use the search form (Figure 2A) to query for data about a specific metabolite, gene (RNA or protein), or reaction in a specific organism. Users can search for data about molecules and reactions by their names, identifiers (e.g. ChEBI id), chemical structures (e.g. InChI representation), and descriptions. Datanator recognizes all organisms in the NCBI Taxonomy database. After submitting the search form, Datanator presents three lists of search results for metabolites, genes, and reactions. Each list is ranked by their relevance to the query (Figure 2B–D). Users can click on each result to obtain tables of measured properties (e.g. concentration, half-life) of the selected molecule or reaction (Figure 2E).

### Advanced searching for clouds of similar measurements

While the amount of data has exploded over the last decade, we still do not have data about every molecule and reaction in every organism and environment. Instead, researchers often impute missing information from data about similar molecules and reactions in related organisms and environments. However, it is difficult to find data about similar biology. For example, searching Google for data about ATP synthase or its orthologs in *E. coli* or other Enterobacteria such as *Klebsiellas* requires combinatorially-many searches for each ortholog in each Enterobacterial species.

To help researchers conduct research when direct measurements are not available, Datanator provides tools for assembling *clouds* of data around a specific molecule or reaction in a particular organism and environment. For metabolites, Datanator can identify measurements of chemically-similar metabolites (Tanimoto coefficient ≥ 0.65). For genes, Datanator can identify measurements of the same OrthoDB (25) ortholog group. For reactions, Datanator can identify measurements of the same Enzyme Commission (EC) class.

Datanator displays these clouds using the data tables described above (Figure 2E). To help users understand these clouds, the data tables include columns that describe the similarity of each measurement to the search query. For metabolites, Datanator displays the Tanimoto coefficient between the target and measured metabolites. Datanator indicates the phylogenetic relevance of each measurement by displaying the taxonomic rank of the most recent canonical-ranked common ancestor between the target and measured organisms (e.g. species, genus).

Datanator also provides users sliders and select menus for narrowing these data clouds to relevant measurements for their work (Figure 2F–H). This includes a slider for filtering measurements of metabolites by the Tanimoto coefficient of the target and measured metabolites, a slider for filtering measurements by the taxonomic distance between the target and measured organisms, sliders for filtering measurements by their temperature and pH, and a select menu for filtering measurements by their growth media.

In addition, Datanator provides users checkboxes for creating data clouds manually (Figure 2E). Researchers can use these checkboxes to create clouds that only include data that they believe is relevant to their work.

Finally, Datanator provides buttons for exporting data clouds to CSV and JSON files.

### Programmatic searching via a REST API

Users can also programmatically query Datanator via a REST API. This can be used to search for data about multiple molecules, reactions, or organisms.

## TOOLS FOR VISUALIZING DATA CLOUDS

To help researchers interpret their data clouds, Datanator also provides tools to chart the distribution of these clouds (Figure 2I). Mouseing over each measurement highlights the measurement in the corresponding data table.

## IMPLEMENTATION

Datanator consists of a database of molecular data, an OpenAPI-compliant REST API for querying the database, and a web application for graphically exploring the database.

### Database construction

We assembled the database in several steps. First, we used Google, Google Scholar and Microsoft Academic to find potential sources of data. Second, for each kind of data, we focused on the largest databases. In the absence of an existing database, we focused on articles that report genome-scale data.

Third, we downloaded and parsed each source. Where available, we used file downloads and APIs. For example, we downloaded PaxDB as an archive of TSV files and we downloaded the Protein Ontology as an OBO file. For databases that do not provide a download or complete API, we wrote scripts to scrape and parse their websites.

Fourth, we normalized each measurement. We normalized the subject of each measurement (molecule or reaction) by mapping it to one of several namespaces. We mapped the structure of each metabolite to InChI, mapped each mRNA and protein to its UniProt id, and mapped each reaction to the InChI representations of its participants. We used BpForms (26) to normalize the representation of each RNA and protein modification profile. We mapped the taxonomic context of each measurement to an NCBI Taxonomy id. We used the pint package to normalize the units of each measurement. Lastly, we combined the measurements into a single MongoDB database.

We implemented the downloading, parsing, normalization, and integration of each data source as a repeatable script.

The database is structured as a warehouse rather than a federated database or registry of datasets for multiple reasons: (a) some of our data sources do not provide APIs, (b) the warehouse design leverages snapshotting to insulate our database from breaking changes to the schemas of the sources, (c) the available APIs for our sources are not all guaranteed to be available in perpetuity and (d) querying a warehouse is faster because the data in the warehouse has already been normalized and integrated.

### Data updating and versioning

Going forward, we aim to periodically re-build the database with the latest version of each source database. This is possible because we have implemented repeatable codes for downloading and integrating each database. We also aim to continue to curate data. Each time we rebuild the database, we plan to increment the version number of the database and archive a snapshot.

### REST API

We implemented the API using Python, connexion, pymongo and Elasticsearch.

### Web application

We implemented the web application using the React framework and several packages, including ag-Grid (data tables), Blueprint (search form) and ChartJS (charts).

### Testing

We tested the database construction and API using unittest. We tested the web application using Cypress and Jest.

## COMPARISON TO EXISTING DATABASES

Numerous resources help researchers find data, including databases for individual types of data and model organism, genome, pathway and federated databases. While several of these resources overlap with Datanator, Datanator uniquely contains multiple types of quantitative and categorical molecular data for multiple organisms. When direct measurements are unavailable, Datanator also provides unique tools for finding data about similar biology.

### Data type-specific databases

Although most of Datanator’s data is available from other databases such as ECMDB and PaxDB, it is challenging to use these databases together. In addition, these databases provide few tools for obtaining data about similar biology. Datanator makes their data more accessible by providing a central, consistent portal to the data. Datanator also provides tools for building data clouds that encompass measurements of similar biology. Additionally, Datanator has more metabolite concentrations than ECMDB or YMDB because it combines ECMDB, YMDB, and several articles.

### Model organism databases

Several model organism databases have overlapping content with Datanator. EcoCyc contains reaction kinetic parameters for *E. coli* (27). SubtiWiki contains RNA and protein expression data for *B. subtilis* (28). However, these databases focus on genetic and relational data, and they each only contain data for one organism. The CCDB (*E. coli*, (29)), MyMPN (*Mycoplasma pneumoniae*, (30)) and WholeCellKB (*M. genitalium*, (7)) contain multiple types of data. Datanator improves upon these databases by incorporating data for multiple organisms and providing more powerful search tools.

### Genome and pathway databases

Several genome and pathway databases also have overlapping content with Datanator. For example, Reactome contains RNA and protein expression data (31) and UniProt contains data about protein modifications (32). However, these databases focus on genetic and relational information, such as the locations of promoters and their regulators. These databases have limited quantitative information, such as concentrations.

### Federated databases

Like Datanator, federated databases such as BioMart (33) and Omics DI (34) are also central portals for discovering multiple types of data. While both databases contain more data than Datanator, their content has little overlap with Datanator. Furthermore, the content of Datanator is more normalized and integrated than that of BioMart and Omics DI. We believe that this normalization and integration makes Datanator more helpful for integrated research.

## USE CASES

Through helping researchers obtain data, we believe that Datanator can assist a wide range of tasks. As outlined below, we believe that Datanator can help researchers conduct comparative analyses of multiple tissues and organisms, conduct multi-dimensional analyses of molecular networks, calibrate mechanistic models, and validate conclusions with independent data. The Supplementary Data describes two case studies of using Datanator to (a) reconstruct the metabolite composition of the cytosol of *E. coli* toward a more detailed biomass reaction for an improved flux balance analysis (FBA) model of its metabolism and (b) analyze the relative stability of mRNA across prokaryotes, archae and eukaryotes to understand the relative metabolic burden of transcription across species.

### Multi-dimensional analyses of biochemical networks

Because Datanator contains multiple types of data, Datanator is a good source of data for analyzing relationships among molecules, reactions, and their chemical properties. For example, Datanator could provide data for analyzing the correlation between the abundances of enzymes and their maximum reaction velocities, classifying reactions as bottlenecks by comparing metabolite concentrations and enzyme-metabolite affinities, or learning a regression model of the half-lives of RNAs from their sequences and modifications.

### Calibrating mechanistic models

For similar reasons, Datanator is also a good source of data for calibrating mechanistic models. For example, Datanator could provide protein abundances to constrain missing initial conditions; provide metabolite concentrations to construct a more comprehensive biomass equation for a FBA model (see example in the Supplementary Data); or provide organism, tissue, or cell-type-specific protein abundances for recalibrating a model to create variants for different organisms or cell types. In particular, Datanator is ideal for integrative modeling of multiple biochemical subsystems such as whole-cell models. For example, Datanator could provide protein abundances and maximum reaction velocities to expand an FBA model to capture gene expression and further constrain the model (5).

### Validating computationally-generated conclusions

In the absence of an experimental collaboration, computational scientists often validate their conclusions with independent data from articles. However, this is often time-consuming because this requires finding data about specific molecules or reactions in particular organisms. By making it easier to find data about specific biology, Datanator empowers computational scientists to follow up their own hypotheses.

## DISCUSSION

In summary, Datanator is a unique portal for obtaining several types of quantitative and categorical molecular data. The foundation of Datanator is an integrated database of several types of data about numerous organisms. This includes the first collection of multiple studies of mRNA half-lives. Datanator is also the first database to contain the concentrations of metabolites in multiple organisms. To help researchers use this data, Datanator provides unique tools for assembling clouds of data centered on specific molecules and reactions in particular genotypes and environments. In addition, Datanator provides charts for visualizing the distribution of these clouds. These clouds can help users obtain informative data even in the absence of direct measurements. We anticipate that Datanator will help researchers build integrative models of multiple biochemical subsystems, including models of entire cells. We believe that Datanator is also a valuable source of data for multi-dimensional analyses of biochemical networks and comparative studies of multiple organisms.

### Data licensing

Our philosophy is provide integrated data with no additional restrictions beyond those imposed by our sources. Unfortunately, several of the most relevant source databases for Datanator state licenses that require attribution and only permit non-commercial usage. Long-term, we hope to encourage databases to be more permissive.

### Community initiative to curate additional data

Going forward, we believe that the most promising way to scalably curate data is for the community to work together. This will require navigating a variety of challenges including developing standards for precisely capturing the semantic meaning of molecular measurements (e.g. which property was measured of which molecule or reaction), getting the community to embrace these standards, developing user-friendly interfaces for contributing data, developing automated quality controls, and creating incentives for the community to contribute. To start, we have developed a single format which is capable of representing any type of molecular measurement. We have also developed a tool for validating that data sets adhere to this format. We organized a meeting during the COMBINE Forum to discuss this format and get feedback from potential collaborators. We also welcome suggestions via GitHub issues and pull requests or by joining our team. Next, we plan to invite the maintainers of a few key databases of molecular data to work with us to integrate their data. We believe this will enable us to both work with a small number of people to incorporate a large amount of already standardized and quality controlled data and get feedback on our data submission process. Ultimately, we hope to be able to accept any type of data from any investigator through a simple form, and then automatically quality control and integrate their data.

### Additional search and analysis tools

To further help users, we plan to develop additional search and analysis tools. For example, we aim to create charts of the global distribution of each type of data; implement a tool for extracting multi-dimensional data clouds such as clouds of the expression and half-lives of mRNA and their proteins; and develop finer-grained filters for data about similar RNAs, proteins, and reactions based on the sequence similarity of RNAs and proteins and the structural similarity of the participants of reactions.

### Higher-level tools for integrative modeling

In addition, we aim to develop higher-level tools for leveraging Datanator for integrative modeling. For example, we aim to build tools for using data from Datanator to constrain the values of missing model parameters, recalibrate models to represent different cell types and further constrain FBA models with enzyme abundances and reaction parameters.

## DATA AVAILABILITY

The application and API are available at https://datanator.info and https://api.datanator.info along with a tutorial and documentation. The copyrightable content of the database curated by the authors is available under the CC0 license; the content aggregated from third-party databases is available under the licenses summarized on the Datanator website. The structure of the database and software tools for working with the database are available under the CC0 and MIT licenses, respectively.

## Supplementary Material

gkaa1008_Supplemental_Files
