# Supplementary material for: Datanator: an integrated database of molecular data for quantitatively modeling cellular behavior
Source: Nucleic Acids Res. 2020 Nov 11;49(D1):D516–22. doi: 10.1093/nar/gkaa1008 (PMC7779073; doi:10.1093/nar/gkaa1008)
Supplement: gkaa1008_Supplemental_Files [file gkaa1008_supplemental_files.zip › Supplementary data.pdf]

Datanator: an integrated database of molecular data for quantitatively modeling cellular behavior: Supplementary Data

Yosef D. Roth*, Zhouyang Lian*, Saahith Pochiraju, Bilal Shaikh and Jonathan R. Karr†

Icahn Institute for Data Science and Department of Genetics and Genomic Sciences, Icahn School of Medicine at Mount Sinai, 1255 5th Avenue, Suite C2, New York, NY 10029, USA.

CASE STUDIES

Below we describe two case studies that illustrate how Datanator can help investigators find data for meta-analyses of multiple studies and comparative analyses of multiple organisms.

A more comprehensive reconstructed cytosolic composition of *Escherichia coli* for an improved FBA model of its metabolism

Data about intracellular metabolite concentrations is needed to help build more predictive models of cellular metabolism. For example, more comprehensive reconstructions of intracellular metabolite concentrations could help constrain Flux-Balance Analysis (FBA) models (1, 2).

Due to the technical limitations, it is difficult to simultaneously characterize the concentration of each metabolite within a single study. As a result, even the most comprehensive studies have only been able to characterize the concentrations of a fraction of the metabolites in a cell. One possible way to assemble a more comprehensive dataset is to integrate data from multiple studies.

One of the best characterized organisms is *Escherichia coli*. Four studies from three research groups have reported metabolome-scale measurements of the concentrations of metabolites in *E. coli* across several growth conditions: Bennett et al., 2009 (3), Gerosa et al., 2015 (4), Ishii et al., 2007 (5), and Park et al., 2016 (6).

Toward a comprehensive reconstruction of the intracellular concentrations of metabolites in *E. coli*, we used the Datanator API to merge these four data sets. First, we extracted the metabolite concentration measurements. Second, we normalized each measurement to mM units. Third, we merged all of the observations associated with each metabolite. Fourth, we computed the mean concentration of each metabolite under glucose-rich media conditions. This resulted in a reconstructed profile of the concentrations of 123 metabolites under glucose-rich conditions. In comparison, the biomass reaction of the most recent FBA model of *E. coli* (7) only captures 70 metabolites. Figure S1a shows the concentrations of the metabolites which were observed in at least one condition in all four studies. Figure S1b shows the distribution of metabolite concentrations measured for each condition. Many of the measurements in glucose-rich media (circles) are significantly different between the data sets, suggesting that more standardization may be needed to facilitate the comparison and integration of metabolomics data.

This reconstructed cytosolic profile could enable a better constrained FBA model by serving as the basis of a more comprehensive biomass equation.

Comparative stability of mRNA from prokaryotes to eukaryotes

Recently, researchers have recognized that most *E. coli* and *Mycoplasma* mRNAs are transiently expressed because prokaryotes have limited mRNA capacity, which must be stochastically rotated among their protein-coding genes. Combined with regulation, unstable mRNAs enable prokaryotes to shift their protein expression quickly. However, the need to frequently replace mRNA is a significant metabolic cost.

To explore whether other prokaryotes and more distant organisms have similar metabolic burdens from unstable mRNAs, we used the Datanator API to gather measurements of mRNA half-lives for the prokaryotes *Bacillus cereus* (8), *Lactococcus lactis* (9), *Mycobacterium smegmatis* (10) and *Mycobacterium tuberculosis* (10); the archaea *Methanosarcina acetivorans* (11); and the eukaryotes *Saccharomyces cerevisiae* (12), *Homo sapiens* (13), and *Mus Musculus* (14). Figure S2 shows the distributions of the mRNA half-lives of each organism. This figure suggests that most prokaryotes, archaea, and single-cell eukaryotes have unstable mRNAs and, consequently, must dedicate significant metabolic resources to transcription. In contrast, mRNAs are much more stable in higher-order eukaryotes, suggesting that transcription is proportionally metabolically-cheaper for these organisms.

---

*The authors wish it to be known that, in their opinion, the first two authors should be regarded as joint first authors.

†To whom correspondence should be addressed. Tel: +1 212-824-9664; Email: karr@mssm.edu

REFERENCES

1. Orth,J. D., Thiele,I. and Palsson,B. Ø. (2010) What is flux balance analysis?. *Nat. Biotechnol.,* **28**, 245–248.
2. Feist,A. M. and Palsson,B. O. (2010) The biomass objective function. *Curr. Opin. Microbiol.,* **13**, 344–349.
3. Bennett,B. D., Kimball,E. H., Gao,M., Osterhout,R., Van Dien,S. J. and Rabinowitz,J. D. (2009) Absolute metabolite concentrations and implied enzyme active site occupancy in Escherichia coli. *Nat. Chem. Biol.,* **5**, 593–599.
4. Gerosa,L., van Rijsewijk,B. R. H., Christodoulou,D., Kochanowski,K., Schmidt,T. S., Noor,E. and Sauer,U. (2015) Pseudo-transition analysis identifies the key regulators of dynamic metabolic adaptations from steady-state data. *Cell Syst.,* **1**, 270–282.
5. Ishii,N., Nakahigashi,K., Baba,T., Robert,M., Soga,T., Kanai,A., Hirasawa,T., Naba,M., Hirai,K., Hoque,A. et al. (2007) Multiple high-throughput analyses monitor the response of E. coli to perturbations. *Science,* **316**, 593–597.
6. Park,J. O., Rubin,S. A., Xu,Y.-F., Amador-Noguez,D., Fan,J., Shlomi,T. and Rabinowitz,J. D. (2016) Metabolite concentrations, fluxes and free energies imply efficient enzyme usage. *Nat. Chem. Biol.,* **12**, 482–489.
7. Monk,J. M., Lloyd,C. J., Brunk,E., Mih,N., Sastry,A., King,Z., Takeuchi,R., Nomura,W., Zhang,Z., Mori,H. et al. (2017) iML1515, a knowledgebase that computes Escherichia coli traits. *Nat. Biotechnol.,* **35**, 904–908.
8. Kristoffersen,S. M., Haase,C., Weil,M. R., Passalacqua,K. D., Niazi,F., Hutchison,S. K., Desany,B., Kolstø,A.-B., Tourasse,N. J., Read,T. D. et al. (2012) Global mRNA decay analysis at single nucleotide resolution reveals segmental and positional degradation patterns in a Gram-positive bacterium. *Genome Biol.,* **13**, R30.
9. Dressaire,C., Picard,F., Redon,E., Loubière,P., Queinnec,I., Girbal,L. and Cocaign-Bousquet,M. (2013) Role of mRNA stability during bacterial adaptation. *PloS One,* **8**, e59059.
10. Rustad,T. R., Minch,K. J., Brabant,W., Winkler,J. K., Reiss,D. J., Baliga,N. S. and Sherman,D. R. (2013) Global analysis of mRNA stability in Mycobacterium tuberculosis. *Nucleic Acids Res.,* **41**, 509–517.
11. Peterson,J. R., Thor,S., Kohler,L., Kohler,P. R., Metcalf,W. W. and Luthey-Schulten,Z. (2016) Genome-wide gene expression and RNA half-life measurements allow predictions of regulation and metabolic behavior in Methanosarcina acetivorans. *BMC Genomics,* **17**, 1–23.
12. Geisberg,J. V., Moqtaderi,Z., Fan,X., Ozsolak,F. and Struhl,K. (2014) Global analysis of mRNA isoform half-lives reveals stabilizing and destabilizing elements in yeast. *Cell,* **156**, 812–824.
13. Duan,J., Shi,J., Ge,X., Dölken,L., Moy,W., He,D., Shi,S., Sanders,A. R., Ross,J. and Gejman,P. V. (2013) Genome-wide survey of interindividual differences of RNA stability in human lymphoblastoid cell lines. *Sci. Rep.,* **3**, 1318.
14. Clark,M. B., Johnston,R. L., Inostroza-Ponta,M., Fox,A. H., Fortini,E., Moscato,P., Dinger,M. E. and Mattick,J. S. (2012) Genome-wide analysis of long noncoding RNA stability. *Genome Res.,* **22**, 885–898.

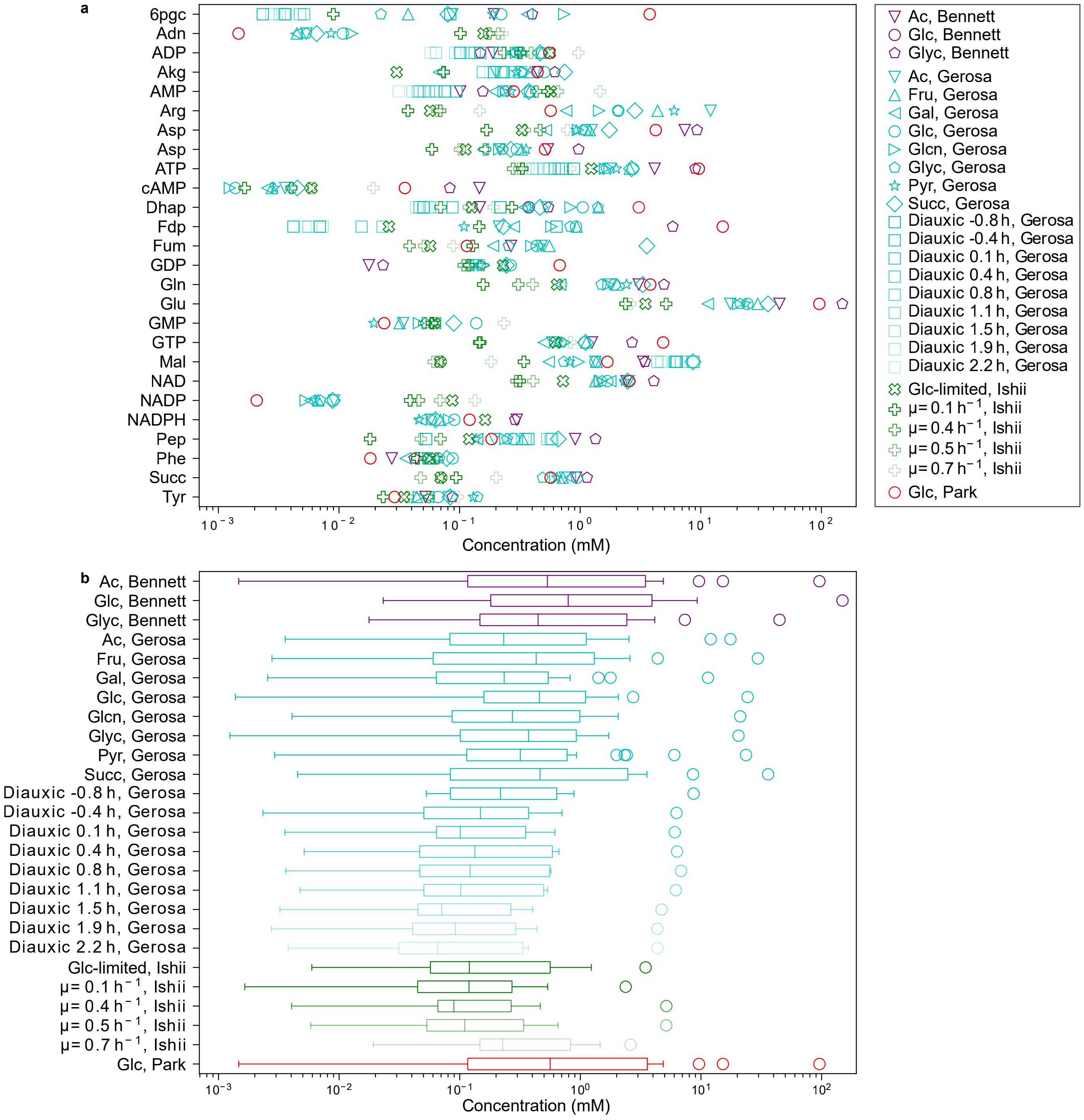

Figure S1. Cytosolic concentrations of 26 metabolites measured in at least one condition reported by Bennett et al., 2009, Gerosa et al., 2015, Ishii et al., 2007, and Park et al., 2016. (**a**) Individual measurements. (**b**) Distributions of the concentrations observed in each condition. The markers indicate the growth condition of each measurement. The colors indicate the datasets. The transparency indicates the time point within the diauxic shift reported by Gerosa et al. and the growth rate series reported by Ishii et al.

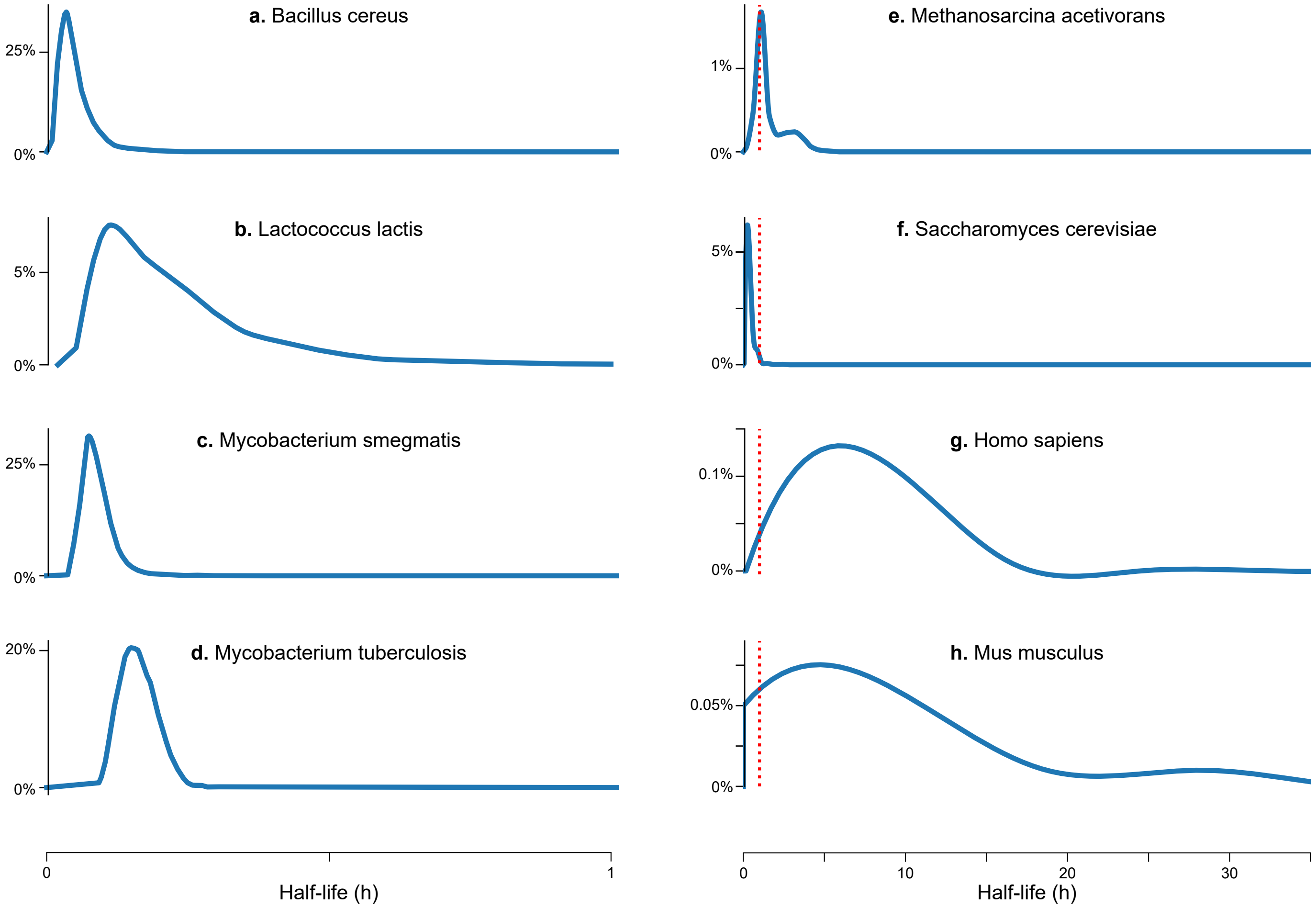

Figure S2. Distributions of the mRNA half-lives of several prokaryotes (left), archaea (top right), and eukaryotes (bottom right). The red lines over the archaea and eukaryote plots indicate the maximum half-lives of the prokaryote plots.
